# High agreement between the new Mongolian electronic immunization register and written immunization records: a health centre based audit

**DOI:** 10.5365/wpsar.2016.7.4.006

**Published:** 2017-09-25

**Authors:** Jocelyn Chan, Tuya Mungun, Narangerel Dorj, Baigal Volody, Uranjargal Chuluundorj, Enkhtuya Munkhbat, Gerelmaa Danzan, Cattram D Nguyen, Sophie La Vincente, Fiona Russell

**Affiliations:** a Pneumococcal Research, Murdoch Childrens Research Institute, Parkville, Australia.; b Department of Paediatrics, University of Melbourne, Parkville, Australia.; c Centre for International Child Health, Murdoch Childrens Research Institute, Parkville, Australia.; d National Center for Communicable Diseases, Ministry of Health, Ulaanbaatar, Mongolia.; e Division for Surveillance and Emergency Operations, Ministry of Health, Ulaanbaatar, Mongolia.; f Immunization unit, National Center for Communicable Diseases, Ulaanbaatar, Ministry of Health, Mongolia.; g Clinical Epidemiology & Biostatistics Unit, Murdoch Childrens Research Institute, Parkville, Australia.

## Abstract

**Introduction:**

Monitoring of vaccination coverage is vital for the prevention and control of vaccine-preventable diseases. Electronic immunization registers have been increasingly adopted to assist with the monitoring of vaccine coverage; however, there is limited literature about the use of electronic registers in low- and middle-income countries such as Mongolia. We aimed to determine the accuracy and completeness of the newly introduced electronic immunization register for calculating vaccination coverage and determining vaccine effectiveness within two districts in Mongolia in comparison to written health provider records.

**Methods:**

We conducted a cross-sectional record review among children 2–23 months of age vaccinated at immunization clinics within the two districts. We linked data from written records with the electronic immunization register using the national identification number to determine the completeness and accuracy of the electronic register.

**Results:**

Both completeness (90.9%; 95% CI: 88.4–93.4) and accuracy (93.3%; 95% CI: 84.1–97.4) of the electronic immunization register were high when compared to written records. The increase in completeness over time indicated a delay in data entry.

**Conclusion:**

Through this audit, we have demonstrated concordance between a newly introduced electronic register and health provider records in a middle-income country setting. Based on this experience, we recommend that electronic registers be accompanied by routine quality assurance procedures for the monitoring of vaccination programmes in such settings.

## Introduction

Monitoring of vaccination coverage is vital for the prevention and control of vaccine-preventable diseases. Coverage estimates are also an important indicator of health system performance and a benchmark for progress towards reducing child mortality. In countries lacking reliable administrative data on vaccinations, the estimation of vaccination coverage relies on conducting vaccination coverage surveys, which are time-consuming and expensive. In addition, conducting such studies requires expertise to prevent selection or information bias. (*1*)

To facilitate the monitoring of vaccination coverage, countries around the world are increasingly adopting electronic immunization registers that are defined as computerised, population-based systems that collect individual-level vaccination data. (*2*) There is strong evidence that the use of immunization registers can increase rates of vaccination. (*3*) They can have an impact at an individual level, assisting health-care providers to ensure that individuals have received the recommended vaccinations, (*4*) and at a population level, highlighting undervaccinated groups to guide vaccination policy. (*5*) Immunization registers are also valuable research tools and can be linked with disease surveillance databases to assess vaccine effectiveness and safety. (*6*)

The usefulness of an immunization register depends on the completeness and accuracy of the information it contains. Several studies in a range of settings have highlighted the potential problem of underreporting of vaccinations, (*7*-*9*) leading to systematically lower coverage estimates. One systematic review reported that out of 17 papers using immunization register data to determine vaccine effectiveness, only one addressed the accuracy of information in the register. (*6*) This highlights the limited literature addressing immunization register data quality despite the need for such studies.

While registers are increasingly widely adopted worldwide, there is limited literature about the use of electronic registers in low- and middle-income country settings such as Mongolia. To coincide with the staged introduction of the 13-valent pneumococcal conjugate vaccine (PCV13), starting with two districts in Mongolia, the national Expanded Programme of Immunizations (EPI) at the Ministry of Health (MoH) developed an electronic immunization register to record PCV13 doses administered. The immunization register allows the EPI team to efficiently monitor vaccination coverage and can be linked with the surveillance system for invasive bacterial and vaccine-preventable diseases (IB-VPD) to monitor vaccine impact. If successful, the MoH plans to expand the immunization register to include all EPI vaccines and to invlove the rest of the country. In this study, we aim to describe the electronic immunization register in Mongolia and determine the completeness and accuracy of PCV13 data to calculate vaccination coverage and determine vaccine effectiveness by comparing electronic records with existing written health provider records.

## Methods

### Description of the electronic immunization register

On 6 June 2016, the Mongolian MoH commenced delivery of PCV13 from all 19 immunization clinics in two districts of the capital city Ulaanbaatar. Infants received PCV13 at 2, 4 and 9 months of age. A catch-up campaign for older children was performed; children aged 3–23 months received two doses at one-month intervals. Immunization nurses documented the following in a registration book: name, national identification (ID) number (unique identification number given at birth), age, address, phone number and date of PCV13 administration. This information was entered into a web-based electronic immunization register at the end of each day. The EPI team, responsible for monitoring the introduction of PCV13, is able to access data from the electronic immunization register in real time.

### Study design

We conducted a cross-sectional chart audit among children 2–23 months of age vaccinated at immunization clinics within the two districts. The main outcome measures for this audit were (1) completeness: the proportion of written records that were able to be identified on the electronic immunization register; and (2) accuracy: proportion of linked records with matching vaccination dates to within seven days.

We used systematic random sampling, selecting the first 32 entries from registration books from each immunization clinic for each month of June, July and August 2016. A sample of 32 per month for each clinic was based on sample size calculated to detect an estimated 80% accuracy with a precision of 2.5% with 95% confidence, taking into account clustering within the 19 immunization clinics using an intra-class correlation of 0.01.

### Data collection

From the registration books, the following data were abstracted: national ID number, district and subdistrict of vaccine administration and PCV13 vaccination dates. These entries were double-entered into Epidata EntryClient v2.0.10.26 (The Epidata Association, Odense, Denmark) and checked for inconsistencies.

The audit was conducted in August 2016 after the completion of the catch-up campaign. The electronic database was exported twice. The first export was one week after collection of the data from the written records on 23 August 2016 and the second was on 3 October 2016 to ensure any delayed data were captured.

### Data analysis

We reviewed the data in the electronic immunization register for internal consistency by describing the proportion of doses that were invalid. We defined vaccination dates as invalid if any dose was dated as given before the date of birth, any dose was dated to have been given after the register was first exported (24 August 2016), the first dose was dated to have been given before the vaccine became available (6 June 2016), the second dose was dated to have been given less than 28 days after PCV13 first became available (4 July 2016), the first dose was dated to have been given at less than 8 weeks of age or the second dose was dated to have been given at less than 12 weeks of age. We reported proportions of invalid doses and reasons.

Data from the written record database were linked with the electronic immunization register using the national ID number. We considered the records from the written registration books to be the gold standard for the purposes of this audit.

We reported completeness and accuracy using proportions and 95% confidence intervals adjusted for clustering within subdistricts. Accuracy was reported using the first export of the electronic database only. Completeness was assessed for both first and second exports to capture and compare timeliness of data entry. We included both valid and invalid doses in the analyses of completeness and accuracy. We reported accuracy by district, subdistrict and month. We completed the analysis using Stata IC 14 (StataCorp LLC, College Station, Texas, USA).

### Ethics

Ethics approval was not sought because this audit was conducted as a part of routine quality assurance in collaboration with the EPI team within the Ministry of Health in Mongolia. No identifying information has been included as part of this report.

## Results

### Total and invalid vaccination doses in the electronic immunization register

From 6 June to 24 August 2016, there were a total of 19 879 doses of PCV13 recorded in the electronic immunization register, including 15 650 first doses and 4229 second doses. Only 87 (0.004%) doses were invalid. The most common reason for a vaccine date being invalid was that the vaccine was recorded as given before the vaccine became available (**Table 1a** and **Table 1b**).

**Table 1a:** Validity of pneumococcal conjugate vaccine dates (first dose), recorded in the electronic immunization register, Mongolia, June–August 2016

		No. vaccine doses (*n* = 15 650)	%
Valid		15 570	99.5
Invalid	Before 6 Jun	43	0.3
	Before birth	24	0.2
	After export	1	0.0
	< 8 wks of age	12	0.1

**Table 1b:** Validity of pneumococcal conjugate vaccine dates (second dose), recorded in the electronic immunization register, Mongolia, June–August 2016

		No. vaccine doses (*n* = 4 229)	%
Valid		4222	99.8
Invalid	Before 1 Aug	2	0.1
	Before birth	2	0.1
	After export	0	0.0
	< 12 wks of age	3	0.1

### Completeness of the electronic immunization register

Of the 1757 records abstracted from written immunization registers, there were 1614 unique IDs (some of the records were different doses for the same patient). The number of records abstracted was slightly less than the intended sample size (*n* = 1824) because some smaller clinics had fewer than 32 doses per month available to abstract. Among the 1614 patients, 1273 were able to be linked using their unique ID to the electronic immunization register abstracted on 24 August 2016, giving the electronic register a completeness of 78.9% (95% CI: 64.7–88.4). For the records that were unable to be linked, we searched the electronic record again on 3 October 2016 and were able to identify 12% additional records, increasing completeness to 90.9% (95% CI: 88.4–93.4).

### Accuracy of the electronic immunization register

For the 1273 patients that were able to be linked, there were 1386 records (or doses) that were able to be compared. The PCV13 dates recorded on the electronic record matched the written record for 93.4% (adjusted 95% CI: 84.1–97.4) of records (**Table 2**). The accuracy of the electronic register was similar by district (**Table 3**). For all but five subdistricts, the proportion of PCV13 vaccine dates from electronic record matched the written record by more than 90% (**Table 4**). The accuracy of the electronic register declined over time (*P* < 0.001) (**Table 5**).

**Table 2:** Accuracy of pneumococcal conjugate vaccine dates recorded in the electronic immunization register, Mongolia, June–August 2016

PCV13 dates		No. records (*n* = 1 386)	%
Match	Exact match	1243	89.7
	Within 7 days	51	3.7
No match		92	6.6

**Table 3:** Accuracy of pneumococcal conjugate vaccine dates recorded in the electronic immunization register by district, Mongolia, June–August 2016

	No. linked records available for comparison (*n* = 1 386)	Accuracy (% with matching dates)
District A	557	94.6
District B	829	92.5

**Table 4:** Accuracy of pneumococcal conjugate vaccine dates recorded in the electronic immunization register by subdistrict, Mongolia, June–August 2016

Subdistrict	No. linked records available for comparison (*n* = 1 386)	Accuracy (% with matching dates)
Subdistrict A	75	100.0
Subdistrict B	89	97.6
Subdistrict C	81	97.5
Subdistrict D	78	91.0
Subdistrict E	89	100.0
Subdistrict F	67	71.6
Subdistrict G	78	100.0
Subdistrict H	82	98.8
Subdistrict I	92	100.0
Subdistrict J	79	96.2
Subdistrict K	84	98.8
Subdistrict L	16	87.5
Subdistrict M	87	100.0
Subdistrict N	33	100.0
Subdistrict O	82	54.9
Subdistrict P	55	83.6
Subdistrict Q	36	86.1
Subdistrict R	89	98.9
Subdistrict S	94	96.8

**Table 5:** Accuracy of pneumococcal conjugate vaccine dates recorded in the electronic immunization register by month, Mongolia, June–August 2016

Month	No. linked records available for comparison (*n* = 1 386)	Accuracy (% with matching dates)
June	461	97.6
July	370	92.4
August	555	90.1

## Discussion

This audit found that the overall completeness of the Mongolian electronic immunization register was high (90.9%; 95% CI: 88.4–93.4) when compared to written records. The increase in completeness between the first export (one week after collection of written records) and the second export (five weeks later) indicates a significant delay in data entry. Any analyses of vaccination coverage should consider this delay. The accuracy of the vaccination dates recorded on the electronic register was also high (93.3%; 95% CI: 84.1–97.4). However, these results should be considered in the context that administrative vaccination data, which we have used as our gold standard, from low- and middle-income countries may not be reliable. (*10*)

Results from different audits and evaluations published in the literature have demonstrated vastly different results, reinforcing the need to validate data from registers before use. Many audits, such as those from the national immunization registers in the United Kingdom, (*11*) Belgium, (*7*) Australia (*8*) and some subnational immunization registers in the United States of America (USA), (*12*, *13*) have demonstrated a high degree of completeness and accuracy with coverage estimates within 10% of coverage estimated from vaccination surveys. (*7*, *8*) However, other audits have demonstrated variability in completeness and accuracy with some noting an improvement in completeness over time from 71.4% to 97.7%; (*14*) others noted an improvement in accuracy from an error rate of 59% to 18% following specific strategies. (*15*)

This audit has demonstrated variability in completeness and accuracy by clinic. Details of underperforming clinics have been passed on to the EPI team for follow-up. While this audit was not designed to determine reasons for poor completeness or accuracy, we anticipate that follow-up visits to underperforming clinics will provide insight into potential issues. The decline in accuracy over time suggests that a process of ongoing monitoring and serial auditing of the registry is needed to maintain quality data. As part of the quality assurance programme for the Norwegian immunization registry, annual reports of children who are incompletely vaccinated or have discrepancies in their schedules are sent to the municipality health services for follow-up. (*16*) Two American registries, in Wisconsin and Philadelphia, noted that completeness and accuracy were greatest among clinics with electronic medical records that linked directly with registry system. (*12*, *13*)

While this audit has validated the data recorded in the immunization register, we have not assessed the quality of the denominator (population) data on which the calculation of accurate vaccination coverage depends. To validate vaccine coverage calculated using administrative data, we recommend conducting a vaccination coverage survey using survey methods recommended by the World Health Organization. (*1*) Our results indicate that the electronic registry can be used to reliably estimate vaccination coverage provided that the denominator data are accurate.

There are several limitations to this study. First, this audit relies on accurate clinic health records for comparison. While we have not reviewed the quality of the clinic data, it is the current source for vaccination coverage estimates and, to our knowledge, the best available source of vaccination information. However, we have also examined completeness using another source – parent-held immunization records. Between November 2016 and February 2017, 569 children recruited as part of enhanced IB-VPD surveillance were noted as having received at least one dose of PCV13 according to their parent-held immunization records. Of these, 86.5% (95%CI: 83.4–89.0) were recorded in the electronic immunization register, indicating similar levels of completeness to our results using clinic data, albeit for a different time period (unpublished data). This process is ongoing. Another potential method to assess vaccination coverage in this population is serosurveys; however, this may not be applicable to pneumococcal conjugate vaccines since there is debate surrounding the reliability of serology as immune correlates of protection. (*17*, *18*)

A second limitation is that we used systematic sampling by month. We chose this method for simplicity to ensure data collection was consistent over the 19 subdistricts. A sample was taken from the beginning of each of the three months, accounting for changes in accuracy from one month compared to another. Therefore, estimates of accuracy are designed to be interpreted over the entire three-month period, and estimates from each month are not reflective of the entire month since the sample was drawn only from the beginning of that month. Third, the study was conducted in an urban setting where the electronic register is being piloted and may not be applicable to other more rural settings. When the electronic register is rolled out country-wide it will be important to re-examine completeness and accuracy of the register. Lastly, our study was not designed to determine reasons for decreases in data accuracy over time; an additional qualitative component may be a useful adjunct.

Immunization registries are increasingly being recognized as important public health tools with both the European Centre for Disease Prevention and Control and United States Centers for Disease Control and Prevention outlining goals to encourage adoption of these systems provided the data entry is timely and accurate. (*19*, *20*) This audit has demonstrated that electronic registers are technically viable in an urban middle-income country setting. This paper describes an effective method for auditing the electronic registers in comparison to health provider records. Comparisons with alternative sources of vaccination data, such as parent-held immunization records, should be considered to triangulate these results given the issues with reliability of administrative data in low- and middle-income countries. (*10*) Based on our experience, we would recommend the adoption of electronic registers, accompanied by routine quality assurance procedures, for the monitoring of vaccination programmes.
